# Reflux symptoms and vocal characteristics in adults with non-organic voice disorders

**DOI:** 10.4102/sajcd.v69i1.935

**Published:** 2022-10-26

**Authors:** Nyasa E. Groenewald, Maria du Toit, Marien A. Graham, Carl Swanepoel, Giselle Maartens, Jeannie van der Linde

**Affiliations:** 1 Department of Speech-Language Pathology and Audiology, Faculty of Humanities, University of Pretoria, Pretoria, South Africa; 2 Department of Science, Mathematics and Technology Education, Faculty of Education, University of Pretoria, Pretoria, South Africa; 3 LCM Voice Clinic, Medical Centre, Groenkloof Life Hospital, Pretoria, South Africa

**Keywords:** acoustic characteristics, laryngopharyngeal reflux, RSI, vocal characteristics, VHI, voice disorders

## Abstract

**Background:**

Laryngopharyngeal reflux (LPR) is prevalent and can lead to voice disorders, but its diagnosis is difficult, because of limited correlations between clinical symptoms and organic pathology. Various tools and methods have been explored to aid a diagnosis of LPR.

**Objective:**

To investigate associations between reflux symptoms, acoustic-, perceptual-, and physical vocal characteristics, glottal function index (GFI), and vocal handicap index (VHI) in adults with non-organic voice disorders.

**Methods:**

Data of 51 adults with non-organic voice disorders were collected, using a retrospective cohort explorative research design, at a private ear, nose and throat specialist practice in Gauteng, South Africa. Quantitative outcomes were compared between reflux symptom index (RSI), acoustic characteristics (jitter, shimmer and fundamental frequency [F0]), maximum phonation time, perceptual- (GRBASI) and physical vocal characteristics, GFI and VHI.

**Results:**

The RSI showed positive fair correlations against GFI, VHI_P_ and caffeine intake, indicating an increase in reflux symptoms with higher scores on the various measures. Moderate correlations were also found between GFI and VHI_P_, grade of hoarseness and jitter, strain and VHI_P_, strain and VHI total (VHI_T_) and between Asthenia and jitter. Very strong correlations were found within the various subsections of the VHI as well as between jitter and shimmer and between F0-male and physical symptoms of the VHI (VHI_P_).

**Conclusion:**

Results indicated associations between reflux symptoms, vocal characteristics, the GFI and the VHI. Based on the correlations found these tools used in conjunction could improve clinical diagnosis of LPR. Implications of these findings are promising, but further research is recommended.

**Contribution:**

This study contributes to the body of knowledge to support the accurate clinical diagnosis of LPR using subjective measures to determine LPR symptoms, as well as acoustic analysis.

## Introduction

Approximately 30% of the global adult population present with a chronic voice disorder (Roy et al., 2005), and more than 50% of patients with voice disorders also present with a form of reflux, more specifically, laryngopharyngeal reflux (LPR) (Kaplan et al., 2014; Koufman et al., 2000, 2002). Another frequent form of reflux, which may be confused with LPR, gastroesophageal reflux disease (GERD), is found in 15% of the global population (Eusebi et al., 2018). A study conducted amongst ear, nose and throat specialists in private healthcare in Gauteng, South Africa reported an LPR incidence rate of 15% (Fourie et al., 2017). Although South African statistics from all healthcare contexts are unavailable, the global prevalence of reflux-related voice disorders supports the need for investigation into this population.

Voice disorders are diagnosed when voice quality, pitch and/or loudness are inappropriate in comparison with individual’s peers of similar age, gender and cultural group (Aronson, 1990; Aronson & Bless, 2009; Boone et al., 2020), and can be classified as organic or non-organic (Voerman et al., 2009). Organic voice disorders are caused by neurological or structural abnormalities. A non-organic voice disorder, on the other hand, is defined as a voice with impaired sound in the absence of causal organic laryngeal pathology (Aronson, 1990; Seifert & Kollbrunner, 2005; Van Thal, 1961; Voerman et al., 2009). Non-organic voice disorders are also frequently referred to as ‘psychogenic’ or ‘functional’ voice disorders (Seifert & Kollbrunner, 2005). These terms are not necessarily interchangeable, but rather fall under the term non-organic voice disorders (Voerman et al., 2009). Certain voice disorders can be caused or worsened by reflux-related disorders, such as LPR (Belafsky et al., 2001; Khan et al., 2006; Ulualp & Toohill, 2009).

Initially, LPR was thought to be a manifestation of GERD (Oyer et al., 2009), but in 1991, it was described for the first time as a disease distinct from GERD (Koufman, 1991). GERD refers to the backflow of contents from the stomach into the oesophagus, whereas LPR refers to the backflow of contents from the stomach, past the oesophagus, into the laryngopharynx (Koufman et al., 2002). LPR and GERD also have distinct clinical differences in terms of patient characteristics, pathophysiology and symptomatology (Belafsky et al., 2002; Johnston et al., 2013). Individuals with GERD experience heartburn, have dysmotility of the oesophagus, are often obese, and while in the supine position their reflux mainly takes place at night (Belafsky et al., 2002). In contrast, individuals with LPR do not experience heartburn, have normal oesophageal motility, and do not tend to be obese, and their reflux occurs mainly during the day (Belafsky et al., 2002). Because of these clinical differences, the outcome measures and diagnostic methods used for GERD are not always suitable for LPR (Belafsky et al., 2002; Johnston et al., 2013).

Even though a distinction has been made between LPR and GERD, LPR is still not well-understood, which complicates its diagnosis and the use of outcome measures (Johnston et al., 2013). The dubiety regarding LPR is partly because of a lack of correlation between the severity of patient symptoms and the severity of the organic pathologies found in the larynx (Belafsky et al., 2001). This discrepancy is caused by vocal symptoms, such as hoarseness and vocal fatigue, being resolved more quickly during treatment than the laryngoscopic findings, such as oedema and mucosal hypertrophy (Belafsky et al., 2001). To further complicate diagnosis, differentiation between LPR and other laryngeal pathologies with the same presentation, as well as the co-occurrence of these conditions, must also be made.

Diagnosing both GERD and LPR can be divided into two approaches: investigation-based and symptom-based. The investigation-based approach is expensive, time-consuming, invasive, not globally available and has certain limitations (Kaplan et al., 2014). Investigation-based diagnosis uses proton pump inhibitor (PPI), endoscopy and pH testing. The investigation-based PPI test used for diagnosing GERD has been found to be controversial for diagnosing LPR (Johnston et al., 2013). Endoscopy methods are limited because of their inability to detect LPR cases, where there are laryngeal lesions without visually perceptible oesophageal involvement (Kaplan et al., 2014). pH monitoring methods are limited as well because of the diagnostic criteria for many pH probe studies having excluded patients who experience acid reflux but lack endoscopic evidence of acid reflux, thus only representing a segment of the range of patients with reflux and resulting in limited accuracy and low sensitivity (60%) of 24 h pH monitoring for these patients (Reynolds, 2007). The symptom-based approach relies on interviews and structured questionnaires (Vakil, 2013); however, up to 50% of patients with extraesophageal symptoms of GERD do not present with typical esophageal symptoms, such as heartburn (Johnston et al., 2013; Vakil, 2013). Therefore, because of the limitations of the investigation-based approach and the uncertainty of symptom-based diagnosis results, new diagnostic methods are needed for LPR.

In an effort to aid the accurate diagnosis of LPR, Belafsky et al. (2002) developed the reflux symptom index (RSI). The RSI showed good criterion-based validity, as it accurately documents symptoms and improvement in patients with LPR (Belafsky et al., 2002). Contradictory findings on the reliability and validity of the RSI have, however, been reported (Hoon Park et al., 2006). To further improve the diagnosis of LPR using the RSI, some researchers have started exploring the possible relationships between the RSI and other vocal characteristics, including hoarseness, breathiness, strain and instability (Lechien et al., 2016; Mesallam et al., 2007). It has also been suggested that the relationship between the RSI and different subgroups of voice disorders should be investigated in order to further improve the precision of its use in diagnosis (Watson et al., 2013). If associations are found between the RSI and certain subgroups of voice disorders, its value as a diagnostic tool may increase. In additional attempts to develop new diagnostic methods, studies have explored the pre- and post-test treatment outcomes in adults with LPR (Karkos et al., 2007; Lechien et al., 2016; Watson et al., 2013). These studies indicated that significant correlations exist between LPR and acoustic and perceptual vocal characteristics, with a significant decrease in jitter, shimmer, vocal handicap index (VHI) and perceptual characteristics (GRBASI) scores 3 month post-treatment of LPR (Lechien et al., 2016; Watson et al., 2013). Another study found an association between LPR and functional dysphonia (a non-organic voice disorder) for two pH parameters (the longest reflux episode in a supine position and the fraction of time the pH was less than four in a supine position) – recommending larger studies on the link between LPR and functional dysphonia (Karkos et al., 2007). This substantiates the need for research to explore the relationships between acoustic, perceptual and physical vocal characteristics (Lechien et al., 2016; Watson et al., 2013) in patients with LPR and non-organic voice disorders. Thus, the research question posed in this study is as follows: What are the associations between reflux symptoms and vocal characteristics (voice quality and voice handicap, as well as patient stroboscopy reports) in adults with non-organic voice disorders?

## Methods

### Study design

A retrospective cohort explorative research design (Sedgwick, 2014) was employed to investigate data sets, case histories, self-rating assessment questionnaires regarding perceived LPR symptoms, voice quality and voice handicap as well as patient stroboscopy reports.

### Setting

Data collection took place from April to July 2019 and was obtained from the existing database of an established clinical ENT, specialising in voice disorders, at a private hospital in Gauteng, South Africa.

### Study population and sampling strategy

Participants were adults aged 18 and older. Individuals with organic pathologies and underlying neurological causes were excluded to ascertain that changes in vocal characteristics were not because of any physical factors. In addition, research has shown that treatment can cause a ‘suggestive effect’ by causing an improvement in one tool (subjective RSI scores) while not in the other (objective reflux finding scores (RFS) [e.g. oedema, oro- and hypopharyngeal erythema and laryngeal keratosis]) (Lechien et al., 2018). Reassessment data could thus affect the correlations and associations between the RSI and other assessment tools. Consequently, to avoid confounding variables, reassessment data sets were excluded and only the data of initial assessments were collected. Only data sets containing a primary diagnosis, vocal fold edge data, a complete RSI, and at least one other complete assessment measure, were included. Some participant data sets included up to three diagnoses – classified as primary, secondary and tertiary – where secondary and tertiary diagnoses specify coexisting conditions other than the main condition affecting the participant’s voice. For example, a participant may have a primary diagnosis of hyperfunctional dysphonia with a secondary diagnosis of LPR. The main diagnoses that were given to participants, based on physical examination, acoustic and perceptual voice analysis as well as self-rating scales, were functional dysphonia, hyperfunctional dysphonia and muscle tension dysphonia (MTD). Hyperfunctional dysphonia and MTD both fall under functional dysphonia, but have progressed enough so as to warrant more specific diagnoses. Hyperfunctional dysphonia is specific to the misuse and abuse of the voice, whereas MTD is specific to a functional dysphonia that specifically involves muscle tension (Van Houtte et al., 2011).

From a database of more than 800 clients, 143 met the inclusion criteria, of which 68 were excluded because of missing or incomplete RSI data sets, and six because of the multi dimensional voice programme (MDVP) having been used with them instead of the Praat programme. Praat is a programme used to analyse and reconstruct recorded speech signals (Boersma & Weenink, 2022). The MDVP and Praat programmes are similar programmes used for the analysis of voice, but individual numerical values obtained by each programme have been shown to vary greatly, making the combined use of results from these programmes inadvisable (Amir et al., 2009). Thus, the data of 51 participants with non-organic voice disorders were analysed.

### Materials and apparatus

The dependent variable was the RSI which measured the subjective reflux symptoms of each patient. The independent variables were gender, age, caffeine intake, physical vocal characteristics, glottal function index (GFI), VHI, jitter, shimmer, fundamental frequency (F0), maximum phonation time (MPT) and perceptual characteristics (GRBASI).

The RSI is a Likert-scale questionnaire consisting of nine questions, as outlined in Table 1 (Belafsky et al., 2002; Lee et al., 2018). Each question is answered by selecting a number from zero to five, ranging from no problem to severe, respectively (Johnston et al., 2013). The total score can be any number between 0 and 45 (Johnston et al., 2013), with a score greater than 13 being considered as abnormal (Belafsky et al., 2002).

**TABLE 1:** Reflux symptom index.

Instructions		No problem	Almost never	Sometimes	A moderate amount	Frequently	Problem is as ‘bad as it can be’
1.	Hoarseness or other voice problems	0	1	2	3	4	5
2.	Clearing throat	0	1	2	3	4	5
3.	Excess throat mucus or postnasal drip	0	1	2	3	4	5
4.	Difficulty swallowing food, liquid or pills	0	1	2	3	4	5
5.	Coughing after eating or after lying down	0	1	2	3	4	5
6.	Breathing difficulties or choking episodes	0	1	2	3	4	5
7.	Troublesome or annoying cough	0	1	2	3	4	5
8.	Sensations of something sticking in throat or lump in throat	0	1	2	3	4	5
9.	Heartburn chest pain, indigestion or stomach acid coming up	0	1	2	3	4	5

*Source*: Belafsky, P.C., Postma, G.N., & Koufman, J.A. (2002). Validity and reliability of the reflux syptom index (RSI). *Journal of Voice, 16*(2), 274–277. https://doi.org/10.1016/S0892-1997(02)00097-8

Within the last month, please rate the following in terms of severity on a six point rating scale.

This study looked at the following physical vocal characteristics: vocal fold edge, glottic closure, mucosal wave, periodicity, phase closure, phase symmetry and vertical level of approximation. The physical vocal characteristics were observed by means of a fibreoptic flexible distal chip optic camera stroboscopy examination. A fibreoptic stroboscopy examination is considered to be the clinical ‘gold standard’ for assessing vocal fold function (Bonilha et al., 2015).

Acoustic characteristics, such as jitter, shimmer and F0, were also analysed. Jitter is the measure of the variation of periodicity in the fundamental frequency of the vibratory characteristics of the vocal folds. A jitter score higher than one is considered abnormal (Schwartz, 2004; Teixeira & Fernandes, 2014). Shimmer is the measure of the variation of periodicity in the amplitude of the vibratory characteristics of the vocal folds, with an abnormal score being anything higher than 5 (Schwartz, 2004; Teixeira & Fernandes, 2014). F0 is the lowest frequency of the voice which correlates with a physical measure of vocal fold vibration, with a score lower than 175 or higher than 230 being considered abnormal for females, and a score lower than 110 or higher than 160 being considered abnormal for males (Ferrand, 2012; Teixeira & Fernandes, 2014).

The GRBASI 4-point rating scale (Yamauchi et al., 2010) is a measurement for the perceptual evaluation of voice quality and is widely used because of its brevity and user-friendliness. It looks at six characteristics of voice quality, including grade of hoarseness (G), roughness (R), breathiness (B), asthenia (A), strain (S) and instability (I). The VHI is a Likert-scale questionnaire which measures the perceived psychosocial effect of voice disorders and consists of 30 items, each of which can be scored between zero and four, with a maximum total score of 120 (Johnston et al., 2013). VHI abnormal scores vary for each subsection. The symptom-focused vocal impairment of each participant was measured using the GFI. The GFI is a self-administered Likert-scale questionnaire which has four questions and is answered by selecting a number from zero to five, with a possible score ranging from 0 to 20 (Johnston et al., 2013), with a score higher than four being considered as abnormal (Bach et al., 2005).

### Data collection

Data collection took place at the voice clinic, where data dating from 2015 to 2019 were extracted from electronic assessment reports and then manually inserted into an MS Excel spreadsheet. The data were then manually coded. The RSI, VHI, GFI and case history were completed subjectively by each client. Flexible distal chip stroboscopy examinations were used by the ENT to conduct objective patient evaluations. In addition, the speech-language therapist (SLT) used the GRBASI and the Praat computer programme to evaluate the patients’ perceptual and acoustic vocal characteristics, respectively.

The ENT and the SLT who conducted the assessments both had more than 10 years of experience in treating voice disorders, ensuring reliable outcomes (Ajmi & Aase, 2021). The cross-check principle was used to ensure the accuracy of measurements. This principle is used to improve accurate diagnosis by cross-checking the results of a single test, in this study the RSI, against an independent test measure; in this case, the reported stroboscopy evaluation outcomes, thus confirming the results of the first test (Hall, 2016; Jerger & Hayes, 1976). This principle was used to account for the reported contradictory findings regarding the RSI’s validity and reliability (Hoon Park et al., 2006). The ENT and SLT reached consensus with the classification (mild, moderate or excessive) of caffeine intake. In addition, bias of evaluation may have influenced GRBASI scores, as the rater had prior knowledge of the patient’s symptoms and history and was, therefore, not blinded during the scoring of the GRBASI.

### Data analysis

The Statistical Package for Social Sciences v24 was used by a statistician to analyse data. Descriptive statistics were calculated for the different variables. For continuous variables, the Shapiro–Wilk test showed a lack of normality; thus, nonparametric tests were used (Field, 2018). The Spearman correlation coefficient (r_s_) was used for correlations between continuous variables; the Chi-square test was used to test for associations between nominal categorical variables. Because of a small cohort and sparse data, the *p*-value of the Fisher’s exact test was used to determine associations instead of the *p*-value of the Chi-square test. The *ad hoc* tests – Phi coefficient and Cramer’s V – were used following the Chi-square test to determine the strength of association. A statistically significant correlation (or association) exists if the *p*-value is less than 0.05 (Field, 2018). Correlations range from -1 to +1; the categorisations of their strength (i.e. poor, fair, moderate, very strong and perfect) were done as per the recommendations of a 2018 user’s guide to correlation coefficients (Akoglu, 2018). A positive correlation indicates that as one variable increases, so does the other, and vice versa, whereas a negative correlation indicates that as one variable increases, the other decreases and vice versa. Phi coefficients and Cramer’s V range from 0 to 1; categorisations of their strength were again done as per the guidelines in the 2018 user guide (Akoglu, 2018). Only statistically significant correlations and associations are reported on. The level of significance and the power of this study are 0.05 and 0.9685, respectively. It should be noted that a statistical power of 0.8 or higher indicates that the sample size was large enough to ensure statistical power. This being said that, prior to data collection, an *a priori* power analysis was conducted using G*Power version 3.1.9.4 (Faul et al., 2007) to compute the required sample size needed for a power of at least 0.8. For conciseness, details are omitted here, as the achieved power was greater than 0.8, indicating that the sample size was sufficient for the tests conducted.

### Ethical considerations

An application for full ethical approval was made to the Institutional Review Board (University of Pretoria Faculty of Health Sciences Research Ethics committee), and ethics consent was received on 27 March 2019, reference number: 1/2019. All data sets used, had previously signed voluntary informed consent forms, giving permission for future research projects to use the data. A permission letter was also obtained from the ENT of the private voice clinic to analyse the existing database. The study was conducted in accordance with the Helsinki Declaration as revised in 2013 (World Medical Association, 2013).

## Results

The data of 51 participants with non-organic voice disorders were analysed, of which 33% were male and 67% were female. Ages ranged from 18 to 77 years (mean = 45.61; standard deviation [SD] = 15.430). No gender or age effect was found. The most prevalent primary diagnoses were MTD (29%) and LPR (28%). Many participants (63%) had either primary, secondary or tertiary diagnosis of LPR, of which 94% had abnormal RSI scores. Only 10% were smokers and 70% were excessive caffeine drinkers (more than 15 cups per week) (Table 2).

**TABLE 2:** Associations of reflux symptom index with demographics of participants.

Participant characteristics	*N*	%	Associations with RSI		
			Statistic	Value of test statistic	*p*
**Primary diagnosis** (*n* = 51)			CramerV	0.493	0.408
Hyperfunctional dysphonia	5	10			
Functional dysphonia	11	21			
Muscle tension dysphonia	15	29			
LPR	14	28			
Other**	6	12			
**Occupation** (*n* = 47)†			Phi	0.690	0.692
Professional voice user	30	64			
Non-Professional voice user	17	36			
**Caffeine intake** (*n* = 40)†			r_s_	0.322	0.043*
Moderate	12	30			
Excessive	28	70			

RSI, reflux symptom index; CramerV, Cramer’s V test; Phi, Phi coefficient; r_s_, Spearman correlation.

† , missing data;

* , statistically significant;

** , for example, non-specific laryngitis, etc.

Table 3 summarises the abnormal descriptive values and results of the RSI, GFI, VHI, jitter, shimmer, MPT and fundamental frequency (F0). Because of separate F0 normative data for males and females, F0 data were analysed according to gender. F0-male showed a positive correlation with the VHI_P_ (r_s_ = 0.874 [very strong]; *p* = 0.005) (Table 3). Positive correlations were found between the RSI and the GFI (r_s_ = 0.366 [fair]; *p* = 0.008), and the RSI and the VHI_P_ subsection (r_s_ = 0.302 [fair]; *p* = 0.035) (Table 3); thus, as the score increased on the GFI or the VHI_P_ subsection, the RSI score also increased. A positive correlation was found between the RSI and caffeine intake (r_s_ = 0.322 [fair]; *p* = 0.043) (Table 2). Most participants with excessive caffeine intake (70%) had abnormal RSI scores (96%).

**TABLE 3:** Abnormal outcomes and Spearman correlations between self-rating and acoustic variables.

Variable		RSI(*n* = 51)		GFI (*n* = 51)		VHI_F_ (*n* = 49)†		VHI_P_ (*n* = 49)†		VHI_E_ (*n* = 49)†		VHI_T_(*n* = 50)†		Jitter (*n* = 33)†		Shimmer (*n* = 33)†		MPT (*n* = 38)†		F0 (*n* = 33)†			
		*n*	%	*n*	%	*n*	%	*n*	%	*n*	%	*n*	%	*n*	%	*n*	%	*n*	%	Male (*n* = 8)†		Female (*n* = 25)†	
																				*n*	%	*n*	%
**Abnorma l**		44	86	46	90	31	63	29	59	29	59	31	62	6	18	11	33	25	66	5	63	15	60
**Norms**		≤ 13		≤ 4		0–9		0–15		0–7		0–30		≤ 1.0		≤ 5		≤ 20		110–160		175–230	
**RSI**	**r_s_**	1.000		0.366		0.164		0.302		0.269		0.263		0.071		0.137		−0.023		0.262		0.214	
	***p***	-		0.008*		0.260		0.035*		0.062		0.065		0.697		0.446		0.154		0.531		0.304	
**GFI**	**r_s_**	-		1.000		0.647		0.761		0.679		0.705		0.400		0.457		−0.509		0.663		0.311	
	***p***	-		-		0.000*		0.000*		0.000*		0.000*		0.021*		0.007*		0.001*		0.073		0.130	
**VHI_F_**	**r_s_**	-		-		1.000		0.875		0.906		0.960		0.463		0.370		−0.325		0.476		−0.107	
	***p***	-		-		-		0.000*		0.000*		0.000*		0.008*		0.037*		0.049*		0.233		0.620	
**VHI_P_**	**r_s_**	-		-		-		1.000		0.926		0.954		0.576		0.439		−0.448		0.874		−0.017	
	***p***	-		-		-		-		0.000*		0.000*		0.001*		0.012*		0.005*		0.005*		0.937	
**VHI_E_**	**r_s_**	-		-		-		-		1.000		0.974		0.483		0.365		−0.401		0.590		−0.056	
	***p***	-		-		-		-		-		0.000*		0.005*		0.040*		0.014*		0.123		0.794	
**VHI**_**T**_	**r_s_**	-		-		-		-		-		1.000		0.510		0.416		−0.423		0.503		−0.035	
	***p***	-		-		-		-		-		-		0.002*		0.016*		0.008*		0.204		0.869	
**Jitter**	**r_s_**	-		-		-		-		-		-		1.000		0.888		−0.427		0.395		0.080	
	***p***	-		-		-		-		-		-		-		0.000*		0.017*		0.333		0.704	
**Shimmer**	**r_s_**	-		-		-		-		-		-		-		1.000		−0.394		0.143		0.192	
	***p***	-		-		-		-		-		-		-		-		0.028*		0.736		0.359	
**MPT**	**r_s_**	-		-		-		-		-		-		-		-		1.000		−0.429		−0.210	
	***p***	-		-		-		-		-		-		-		-		-		0.337		0.325	

RSI, reflux symptom index; GFI, glottal function index; VHI, voice handicap index; VHI_F_, functional VHI subscale; VHI_P_, physical VHI subscale; VHI_E_, emotional VHI subscale; VHI_T_, VHI total; MPT, maximum phonation time; F0, fundamental frequency; r_s_, Spearman correlation.

† , Missing data;

* , statistically significant.

All the correlations between jitter, shimmer, MPT, GFI and VHI were positive, except for correlations with MPT, which were negative (Table 3). The strongest of these correlations were between: VHI_P_ and VHI_E_ (r_s_ = 0.926 [very strong]; *p* < 0.001), VHI_F_ and VHI_E_ (r_s_ = 0.906 [very strong]; *p* < 0.001), VHI_F_ and VHI_P_ (r_s_ = 0.875 [very strong]; *p* < 0.001), jitter and shimmer (r_s_ = 0.888 [very strong]; *p* < 0.001) and between GFI and VHI_P_ (r_s_ = 0.761 [moderate]; *p* < 0.001).

The mean for each of the GRBASI sections ranged from 0.76 to 1.29 (0 = normal; 1 = slight pathology). All correlations between the GRBASI and RSI, GFI, VHI, jitter, shimmer and MPT can be found in Table 4. The strongest correlations were: G and jitter (r_s_ = 0.755 [moderate]; *p* < 0.001), S and VHI_P_ (r_s_ = 0.754 [moderate]; *p* < 0.001), S and VHI_T_ (r_s_ = 0.714 [moderate]; *p* < 0.001) and A and jitter (r_s_ = 0.704 [moderate]; *p* < 0.001) (Table 4).

**TABLE 4:** Spearman correlations of the GRBASI (*n* = 41)† with self-rating and acoustic variables.

Variable	G				R				B				A				S				I			
	*N*	%	r_s_	*p*	*N*	%	r_s_	*p*	*N*	%	r_s_	*p*	*N*	%	r_s_	*p*	*N*	%	r_s_	*p*	*N*	%	r_s_	*p*
**0 (normal)**	9	22	-	-	14	34	-	-	21	51	-	-	19	46	-	-	14	34	-	-	4	10	-	-
**1 (slight)**	18	44	-	-	19	46	-	-	12	29	-	-	15	37	-	-	17	41	-	-	25	61	-	-
**2 (moderate)**	7	17	-	-	7	17	-	-	4	10	-	-	5	12	-	-	6	15	-	-	11	27	-	-
**3 (severe)**	7	17	-	-	1	3	-	-	4	10	-	-	2	5	-	-	4	10	-	-	1	2	-	-
**RSI**	-	-	−0.007	0.966	-	-	−0.096	0.549	-	-	0.143	0.371	-	-	0.028	0.086	-	-	0.078	0.628	-	-	0.160	0.317
**GFI**	-	-	0.461	0.002*	-	-	0.105	0.513	-	-	0.415	0.007*	-	-	0.372	0.017*	-	-	0.543	0.000*	-	-	0.439	0.004*
**VHI**_**F**_	-	-	0.565	0.000*	-	-	0.326	0.040*	-	-	0.502	0.001*	-	-	0.658	0.000*	-	-	0.676	0.000*	-	-	0.490	0.001*
**VHI**_**P**_	-	-	0.598	0.000*	-	-	0.374	0.017*	-	-	0.544	0.000*	-	-	0.653	0.000*	-	-	0.754	0.000*	-	-	0.551	0.000*
**VHI**_**E**_	-	-	0.586	0.000*	-	-	0.367	0.020*	-	-	0.548	0.000*	-	-	0.638	0.000*	-	-	0.689	0.000*	-	-	0.493	0.001*
**VHI**_**T**_	-	-	0.595	0.000*	-	-	0.352	0.024*	-	-	0.556	0.000*	-	-	0.669	0.000*	-	-	0.714	0.000*	-	-	0.519	0.001*
**Jitter**	-	-	0.755	0.000*	-	-	0.588	0.000*	-	-	0.644	0.000*	-	-	0.704	0.000*	-	-	0.654	0.000*	-	-	0.496	0.003*
**Shimmer**	-	-	0.662	0.000*	-	-	0.521	0.002*	-	-	0.605	0.000*	-	-	0.637	0.000*	-	-	0.536	0.001*	-	-	0.432	0.012*
**MPT**	-	-	−0.408	0.011*	-	-	−0.122	0.465	-	-	−0.483	0.002*	-	-	−0.364	0.025*	-	-	−0.441	0.006*	-	-	−0.381	0.018*

G, Grade of hoarseness; R, Roughness; B, Breathiness; A, Asthenia; S, Strain; I, Instability; RSI, reflux symptom index; r_s_, Spearman correlation; GFI, glottal function index; VHI, voice handicap index; VHI_F_, functional VHI subscale; VHI_P_, physical VHI subscale; VHI_E_, emotional VHI subscale; VHI_T_, VHI total; MPT, maximum phonation time.

† , missing data;

* , statistically significant.

According to physical examination results, 16% showed open phase predominates for phase closure, whereas 84% showed normal phase closure. A positive correlation was found between the RSI and phase closure (r_s_ = 0.424 [fair]; *p* = 0.035), indicating that patients with normal phase closure scored low on the RSI and those with abnormal phase closure scored high on the RSI (Table 5).

**TABLE 5:** Associations of RSI with physical vocal characteristics.

Physical examination	Characteristic	*N*	%	Associations with RSI		
				Statistic	Value of test statistic	*p*
**Vocal fold edge-left (*n* = 51)**	Smooth	43	84	r_s_	0.114	0.427
	Smooth, mucus on vocal cord	8	16			
**Vocal fold edge-right (*n* = 51)**	Smooth	48	94	r_s_	0.162	0.257
	Smooth, mucus on vocal cord	3	6			
**Glottic closure (*n*= 50)†**	Complete closure	12	24	r_s_	0.107	0.459
	Incomplete closure	38	76			
**Mucosal wave-left (*n* = 32)** †	Normal	23	71	CramerV	0.074	0.235
	Decreased	4	13			
	Increased	1	3			
	No visible wave	4	13			
**Mucosal wave-right (*n* = 32)** †	Normal	23	71	CramerV	0.074	0.235
	Decreased	4	13			
	Increased	1	3			
	No visible wave	4	13			
**Periodicity (*n* = 16)** †	Regular	15	94	r_s_	0.310	0.242
	Irregular	1	6			
**Phase closure (*n* = 25)** †	Normal	21	84	r_s_	0.424	0.035*
	Open phase predominates	4	16			
**Phase symmetry (*n* = 27)** †	Regular	23	85	r_s_	−0.007	0.974
	Irregular	4	15			
**Vertical level of approximation (*n* = 33)** †	Equal	32	97	r_s_	0.158	0.380
	Questionable	1	3			

RSI, reflux symptom index; r_s_, Spearman correlation; CramerV, Cramer’s V test.

† , missing data;

* , statistically significant.

## Discussion

### Comparisons with other studies

This study explored the associations between LPR symptoms, vocal characteristics and patient-completed questionnaires, to better understand the characteristics and diagnosis of LPR. More than half of the participants (63%) had a primary, secondary or tertiary diagnosis of LPR. The analysis of the associations between RSI, MPT, VHI, GFI, acoustic-, perceptual-, and physical vocal characteristics produced various significant correlations.

Correlations between the GRBASI and other variables varied from fair to moderate. The strongest of these was between G and jitter (r_s_ = 0.755 [moderate]; *p* < 0.001). Other studies have found similar results, and the correlation between jitter and G is the most constant finding when comparing acoustic variables with GRBASI (Lechien et al., 2016; Ziwei et al., 2014). This might be explained by the close relationship between jitter and the stability of mucosal movement of the vocal cords, which is also affected by airflow and amount of mucous on the vocal cords, which in turn is linked to hoarseness of voice (Jin et al., 2008). Thus, an improvement in hoarseness of voice should reflect in the jitter.

Other correlations with jitter were all fair except between jitter and shimmer (r_s_ = 0.888 [very strong]; *p* = 0.005). These results confirm recent findings for correlations between jitter and shimmer (Karlsen et al., 2018; Ziwei et al., 2014), and between jitter and VHI_P_ (Dehqan et al., 2017; Karlsen et al., 2018; Ziwei et al., 2014). The correlations, between perturbation (jitter and shimmer) and VHI_P_, indicate that there is a common underlying entity, that is, vocal fatigue associated with functional voice disorders, between them (Karlsen et al., 2018; Ziwei et al., 2014).

A positive correlation was found between RSI scores and caffeine intake VHI_P_ and F0-male (r_s_ = 0.874 [very strong]; *p* = 0.005), but all other significant correlations between the VHI and acoustic variables were fair. This may be because of the non-organic selection criteria of this study, as research has indicated that associations are not strong between VHI scores and acoustic parameters in participants without direct vocal cord disease (Dehqan et al., 2017; Karlsen et al., 2018; Schindler et al., 2009). Further research, exploring acoustic scores and subjective parameters across populations with specific vocal pathologies, is needed (Karlsen et al., 2018; Ziwei et al., 2014).

A positive correlation was found between high RSI scores and excessive caffeine intake (r_s_ = 0.322 [fair]; *p* = 0.043), indicating that participants with excessive caffeine intake had higher RSI scores. Although some studies have shown that caffeine intake may be related to reflux (Pehl et al., 1997), more recent literature has found this data weak (Katz et al., 2013; Kroch & Madanick, 2017). The relationship between caffeine and the RSI specifically has not been explored to the authors’ knowledge.

The analysis of the associations between reflux symptoms and acoustic-, perceptual-, and physical vocal characteristics, GFI and VHI produced three statistically significant fair correlations. Correlation between the RSI and physical vocal characteristic phase closure was positive, indicating that patients with abnormal phase closure were more likely to have reflux than those with normal phase closure (r_s_ = 0.424 [fair]; *p* = 0.035). No other studies have explored this relationship and further research is, therefore, necessary. The correlation between RSI and VHI_P_ was positive (r_s_ = 0.302 [fair]; *p* = 0.035), indicating a link between patients’ perception of their physical voice symptoms and of their reflux symptoms. Similar correlations were reported, showing a positive correlation between improved RSI and VHI scores after treatment (Belafsky et al., 2002). In addition, a positive correlation was found between the RSI and the GFI (r_s_ = 0.366 [fair]; *p* = 0.008), indicating a link between glottal dysfunction and increased reflux. This presents the possibility of the RSI being used in conjunction with the GFI when evaluating LPR. This relationship has also not been explored in the past, perhaps because of the GFI having been created to assess glottal dysfunction and not specifically LPR (Bach et al., 2005). Thus, if further research confirms these correlations as meaningful, the RSI may be used with the GFI and VHI for more reliable diagnosis of reflux-related voice disorders. In future studies, objective clinical testing should be included so as to ensure that unbiased results are obtained for comparison. Currently, however, empirical treatment is considered best practice for the diagnostic confirmation of reflux-related voice disorders (Falk & Vivian, 2016). Reported associations between the RSI and vocal characteristics may be an indicator of the individuals who would benefit from empirical diagnostic testing.

Correlations of moderate strength were found between the GFI and the VHI_P_, VHI_T_, VHI_E_ and the VHI_F_. Little research is available on the relationship between the GFI and VHI other than that by Bach et al. (2005), who found a correlation of 0.61 (*p* < 0.001) between total GFI and VHI scores, which significantly reduced post-therapy for organic voice disorders. However, albeit not the aim of the current study, these significant correlations indicate that although these are two distinct clinical tools, they may both be used in conjunction with the RSI to increase reliability in monitoring vocal pathology. As significant correlations were found between the RSI and VHI_P_ and between the VHI and GFI, these relationships should be further explored to establish whether the RSI, GFI and VHI have meaningful significant relationships, which could make the diagnosis of LPR using subjective-based instruments more certain.

### Clinical applicability of the study

The results of this study indicate that associations exist between reflux symptoms and vocal characteristics, but further research is necessary to determine whether these results hold any true value for improving the diagnostic criteria for LPR.

### Strengths and limitations

Because of the strict exclusion criteria, confounding variables were limited and results were very specific. All data measures that were included are applicable to the typical voice practice. In addition, this study also has a high achieved power (0.9685) with a high power (typically 0.8 or greater), indicating that there is a large chance of a significance test detecting a true correlation or association. Limitations of the study include the strict exclusion criteria and retrospective nature of this study made it necessary to exclude participants who met the requirements, but did not have complete RSI forms, and may have led to selection bias. The cohort size (*n* = 51) of this study was smaller than expected. Only a minority of people seek private healthcare services for their voice disorders, even though its subjective impact on their lifestyles may be significant in terms of vocational demands and social interaction (Bhattacharyya, 2014). Furthermore, Fourie et al. (2017) found that the incidence of voice disorders amongst private practices in Gauteng specifically was only 5.2% (Fourie et al., 2017). It can be expected that the prevalence of voice disorders is much higher than the incidence rate but that very few people seek treatment (Fourie et al., 2017), often because of time and leave constraints, as well as a lack of awareness of when to seek help and whom to seek help from (Da Costa et al., 2012). An international study reported that people often seek help only when it severely affects their ability to perform their vocational duties (Roy et al., 2005). This, together with the strict exclusion criteria, may explain the small cohort, which limited the types of statistical tests that could be used for data analysis.

## Conclusion

This study found significant correlations between the RSI and phase closure, GFI, VHI_P_ and caffeine intake. These correlations, however, were not very strong and require further exploration. Various correlations between the jitter, shimmer, F0-male, GRBASI, MPT, GFI and VHI were found, varying from poor to very strong. These results support existing research and indicate that there are underlying associations between reflux symptoms and vocal characteristics in adults with non-organic voice disorders. Further investigation is needed to establish the degree of significance of these findings. To determine whether the RSI, phase closure, the GFI and VHI_P_ would improve the accuracy of the diagnosis of LPR, future research would have to compare these parameters within different subgroups of voice disorders, such as functional dysphonia, instead of within the broad group of non-organic voice disorders. This would allow the researcher to determine whether results are specific to certain voice disorders, improving the precision of its use in diagnosis. The manner of diagnosis of LPR for the study would also have to exclude the RSI entirely, so that correlations would not be biased towards the RSI.
